# Topical and Intralesional Immunotherapy for the Management of Basal Cell Carcinoma

**DOI:** 10.3390/cancers16112135

**Published:** 2024-06-04

**Authors:** Aurora Fernández-Galván, Pedro Rodríguez-Jiménez, Beatriz González-Sixto, María Teresa Abalde-Pintos, Beatriz Butrón-Bris

**Affiliations:** 1 Dermatology Department, Hospital Universitario La Princesa, Diego de León St. 62, 28006 Madrid, Spain; aurora.fernandez@salud.madrid.org (A.F.-G.); beatriz.butron@salud.madrid.org (B.B.-B.); 2 Dermatology Department, Hospital Ruber Internacional, 28034 Madrid, Spain; 3 DIPO Research Group, Galicia Sur Health Research Institute (IIS Galicia Sur), SERGAS-UVIGO, 36213 Pontevedra, Spain; beatriz.gonzalez.sixto@sergas.es (B.G.-S.); teresa.abalde.pintos@sergas.es (M.T.A.-P.)

**Keywords:** basal cell carcinoma, BCC, skin cancer, non-surgical treatment, topical immunotherapy, BCC treatment

## Abstract

**Simple Summary:**

Basal Cell Carcinoma is the most prevalent cancer in the white population, with a 30% lifetime risk for individuals with fair skin. The main treatment goal is to completely remove the tumor while preserving function and appearance. Surgery is usually the preferred method due to its high success rates and the ability to verify that the entire tumor has been excised. However, topical and intralesional immunotherapy, although traditionally secondary to surgery, have demonstrated effectiveness and good cosmetic outcomes. These non-surgical treatments are valuable alternatives for patients who cannot undergo surgery. The objectives of this review are to summarize the current state of topical and intralesional immunotherapy treatments for BCC and to evaluate their potential as effective alternatives for certain patient populations.

**Abstract:**

Basal Cell Carcinoma (BCC) is the most common type of cancer among the white population. Individuals with fair skin have an average lifetime risk of around 30% for developing BCC, and there is a noticeable upward trend in its incidence rate. The principal treatment objectives for BCC involve achieving the total excision of the tumor while maximizing the preservation of function and cosmesis. Surgery is considered the treatment of choice for BCC for two main reasons: it allows for the highest cure rates and facilitates histological control of resection margins. However, in the subgroup of patients with low-risk recurrence or medical contraindications for surgery, new non-surgical treatment alternatives can provide an excellent oncological and cosmetic outcome. An evident and justified instance of these local therapies occurred during the COVID-19 pandemic, a period when surgical interventions carried out in hospital settings were not a viable option.

## 1. Introduction

Basal Cell Carcinoma (BCC) is the most common type of cancer among the white population. Individuals with fair skin have an average lifetime risk of around 30% for developing BCC, and there is a noticeable upward trend in its incidence rate [1,2]. The primary carcinogenic factor is exposure to ultraviolet radiation, and it tends to occur more commonly on sun-exposed areas of the skin [3].

Several variants of typical BCC, including superficial, nodular, morphoeic, and ulcerated, are clinically identified. Nevertheless, common BCCs exhibit significant polymorphism and can be challenging to categorize within these established subtypes [1]. It is important not to universally label BCC as “indolent cancer”; such a reputation is warranted only when they are promptly and effectively treated. Although rarely leading to metastatic disease, it can cause significant morbidity related to invasion and destruction of neighboring anatomical structures [4].

The principal treatment objectives for BCC involve achieving the total excision of the tumor while maximizing the preservation of function and cosmesis. Surgery is considered the treatment of choice for BCC for two main reasons: it allows for the highest cure rates and facilitates histological control of resection margins. However, in the subgroup of patients with low-risk recurrence or medical contraindications for surgery, new non-surgical treatment alternatives can provide an excellent oncological and cosmetic outcome [3]. An evident and justified instance of these local therapies occurred during the COVID-19 pandemic, a period when surgical interventions carried out in hospital settings were not a viable option [5].

Within non-surgical therapeutic modalities, the aim of this article was to review the literature associated with topical and intralesional immunotherapy for the management of BCC.

## 2. Materials and Methods

An exhaustive literature search was conducted using PubMed and Cochrane Library. The search was limited to articles in English and Spanish that evaluated topical and intralesional treatments in BCC, employing predefined keywords. All types of studies were included, although meta-analyses, systematic reviews, and observational studies were preferably selected. We did not analyze the staging as this information was not available in most cases.

## 3. Clinical Scenarios and Indications for the Use of Topical and Intralesional Immunotherapy in Basal Cell Carcinoma

The primary determinant in the selection of a medical therapy for BCC is centered on evaluating its risk of local recurrence. Multiple factors come into play when classifying a BCC as either a high- or low-risk tumor for recurrence (location, size, well or poorly defined margins, histological subtype, or previous recurrences). 

In standard clinical practice, in accordance with the main therapeutic guidelines, topical therapies, particularly immunotherapy, are typically reserved for cases of superficial BCC (sBCC), considered low-risk. Additionally, they may be considered for nodular BCC (nBCC) declining surgical intervention or for whom surgery is contraindicated due to patient-related factors such as age, comorbidities, medications, or logistical challenges [6]. In Europe, there are two approved topical agents for sBCC, specifically, 5% imiquimod and topical five percent 5-fluorouracil (5-FU) [2].

### 3.1. Topical Imiquimod

The European Medicines Agency (EMA) has granted approval for the use of imiquimod 5% cream in the treatment of small Basal Cell Carcinomas (slow-growing types of skin cancer). The recommended regimen for BCC is to apply the cream five times a week for six weeks. It is postulated that imiquimod exerts its effects by modulating the immune system’s response to neoplastic cells. Specifically, it acts by stimulating Toll-like receptors 7 and 8, thereby triggering a type 1 helper T cell cytokine response [7]. 

Numerous studies not only confirm the satisfactory efficacy of imiquimod in achieving histological clearance of BCC, in both sBCC and nBCC [8], but also establish its non-inferiority and effectiveness when compared to alternative topical therapies. For instance, in a study, 601 patients received three treatments: methyl aminolevulinate photodynamic therapy (MAL-PDT), imiquimod cream (once daily, 5 times a week for 6 weeks), or fluorouracil cream (twice daily for 4 weeks). Success rates were 72.8%, 83.4%, and 80.1%, respectively. Imiquimod showed superiority over MAL-PDT and similarity to fluorouracil in treating superficial Basal Cell Carcinomas [9,10].

Compared to surgery, Williams et al. discovered that after 5 years, imiquimod had a success rate of 82.5%, whereas surgery achieved a higher success rate of 97.7% [10].

Furthermore, the documented adverse effects, including erythema, scabbing, itching, pain, and tenderness, are consistently reported to be within acceptable limits [10,11].

Imiquimod 5% cream, despite showing lower efficacy compared to surgery, emerges as a reasonable option for treating small, low-risk sBCC. This choice depends on factors such as patient preference, lesion size and location, and whether the patient has multiple lesions [12].

### 3.2. 5-Fluorouracil

Currently, 5-FU can be administered either topically or through intralesional infiltration. For decades, topical 5-FU cream has received approval from major drug regulatory agencies (FDA and EMA) for treating low-risk sBCC. Its effectiveness is extensively documented and supported by numerous studies and clinical trials. However, there has been limited research on the use of intralesional 5-FU for BCC. The first study on this approach was conducted by Müller et al. in 1997, demonstrating positive results. Despite lacking official approval, intratumoral 5-FU has been used off-label for both nBCC and sBCC since then [13,14,15].

5-FU is a pyrimidine analogue known for its ability to disrupt DNA and RNA synthesis by inhibiting the enzyme thymidylate synthetase. Acting as a cytostatic agent, it impedes the incorporation of purines and pyrimidines into DNA during the cell cycle, thereby arresting cell growth and inducing cell death [9,16].

Topical 5-FU cream, typically administered at a 5% concentration, is usually applied twice daily for durations ranging from 2 to 12 weeks. Despite its higher recurrence rates compared to conventional surgery, topical 5-FU demonstrates remarkable efficacy in achieving complete tumor clearance and cure rates at 5 years, reaching up to 90% in some studies [6,16,17]. Side effects associated with 5-FU cream are similar to those observed with imiquimod cream, with the severity and occurrence varying among patients and depending on their compliance with treatment. These side effects include local reactions such as pain, itching, a burning sensation, erythema, alterations in skin pigmentation, crusting, and even blister development, while systemic reactions are less common [18,19,20].

Comparative studies suggest that 5-FU may be less effective than imiquimod when used as monotherapy for low-risk BCC. While 5-FU exhibits higher recurrence rates at 3 and 5 years, similar cosmetic outcomes and a slight reduction in side effects have been observed. There is a slight decrease in pain and local reactions, as well as fewer flu-like systemic reactions, compared to imiquimod [6,12,18,20,21]. Conversely, 5-FU cream appears to be superior to photodynamic therapy (PDT) in terms of the risk of recurrences at 3 and 5 years of follow-up, with no differences in aesthetic outcomes and less pain during treatment [12,21].

There are no consensus guidelines regarding the optimal treatment regimen for intralesional 5-FU. The trial conducted by Miller et al. in 1997 showed slightly lower rates of early recurrence in low-risk BCC when using a regimen of 1 mL twice weekly for three weeks compared to other regimens [12,14]. Various studies in the literature have investigated intralesional 5-FU for both low- and high-risk BCC, using volumes and doses ranging from 0.5 to 2 mL at concentrations between 15 and 50 mg/mL, administered 1–3 times weekly for 2–6 weeks. These studies have shown complete cure rates at 9–12 weeks exceeding 90%. Local and transient effects such as edema, erosion, and necrosis are the most common side effects observed, while systemic effects (cytopenias and gastrointestinal disturbances) are rare. Clinical trials aimed at evaluating the safety and efficacy of intralesional 5-FU to regulate dosage and obtain approval are currently in the recruitment phase (NCT06150144) [12,18,22,23].

### 3.3. Photodynamic Therapy

Topical PDT has indications and has been effectively utilized in the treatment of non-melanoma skin cancer (NMSC), although its use in dermatology has recently extended beyond cancer to include inflammatory conditions and even cosmetic skin rejuvenation procedures. For BCC, it is approved for treating low-risk BCC in many countries, although its off-label use is observed in some, like the USA [2,24].

The exogenous photosensitizers used in PDT include 5-aminolevulinic acid (ALA) and methyl aminolevulinate (MAL), with hexylaminolevulinate being a more recent addition, showing promising results. There are differences in concentrations, regimens, timing, type of agent used, or previous treatment with debulking curettage among countries and physicians. ALA is used more in the USA, while MAL is more common in Europe and Australia. The approved protocol for MAL PDT consists of two sessions of PDT with 20% MAL cream, spaced one week apart. The cream is applied to the tumor and surrounding skin and covered for 2–3 h. During this period, the photosensitizers accumulate in rapidly proliferating cells and act as prodrugs, undergoing metabolism to produce protoporphyrin IX. Upon stimulation and activation by red light, protoporphyrin IX generates cytotoxic oxygen species and free radicals, ultimately leading to cell destruction [12,17,25].

Adverse effects observed with PDT include itching and pain during radiation, along with localized reactions like erythema, weeping, crusting, or blistering at the application site. Less common effects may include hyperpigmentation, tissue necrosis, or scarring. Severity and occurrence vary among patients and depend on factors such as PDT technique and the concentration of the photosensitizer agent used [12].

Clinical trials have shown cure rates with MAL PDT ranging from 60 to 100%, with better outcomes observed in patients with sBCC compared to nBCC or high-risk BCC. However, the 5-year recurrence risk can reach 22%, a figure that tends to be higher in real-world studies, where 5-year cure rates hover around 60%. Nonetheless, due to the lack of histological controls, concerns about residual disease at the treatment site arise. Therefore, PDT for BCC is recommended to be repeated after 1 to 4 weeks and to undergo multiple cycles to ensure complete healing and decrease the risk of recurrence [2,6,12].

Comparative studies with conventional surgery reveal poorer tumor control but excellent cosmetic outcomes with PDT. Comparative studies with other non-surgical interventions demonstrate lower cure rates and higher recurrence rates with PDT compared to imiquimod but similar or not superior outcomes compared to 5-FU. All these treatments achieve comparable cosmetic results [2,6,9,12]. Additionally, subgroup evaluations have shown better outcomes with PDT compared to other therapies in lesions on the lower limbs in older patients [26]. Comparative studies between ALA and MAL have also been conducted, which show no differences in cure rates and recurrence rates between the two [27].

Less local inflammatory effects and fewer moderate to severe reactions were observed in PDT-treated patients compared to those treated with 5-FU and imiquimod. However, transient local reactions such as pain and a burning sensation were more frequently reported with PDT than with conventional surgery [6,12,16,17].

In recent years, intralesional PDT has emerged as a novel approach in dermatology. While its research focus seems to be advancing in hidradenitis suppurativa and acne, promising results have also been demonstrated in the treatment of high-risk BCC, where conventional PDT may fall short due to limited penetration of the photosensitizer and light.

Protocols typically entail using 1% ALA in saline or lyophilized solution, administered at doses ranging from 0.2 to 1 mL/cm^2^. Following application, occlusion is maintained for 2–3 h, and the light is administered under local anesthesia, either intratumorally or through external irradiation. Encouragingly, complete clearance has been achieved in small case series and comparative studies, showcasing non-inferiority to surgery with just a single session of intralesional PDT and no debulking required. While there have been no significant differences noted between external irradiation and intratumoral application, the latter has exhibited slightly higher response rates. The procedure has led to favorable aesthetic outcomes, with initial necrosis well tolerated and resolving within approximately 4–6 weeks [28,29].

### 3.4. Ingenol Mebutate

Ingenol mebutate (IM) gel, derived from the natural extract of the *Euphorbia peplus* plant, is a therapy with chemotherapeutic effects not clearly established but with promising efficacy results in skin cancer. Currently, it is approved by the FDA and other countries solely for the field treatment of actinic keratosis (AK). However, it should be noted that it is no longer commercially available. In BCC, it is used off-label in experimental studies and isolated cases of superficial and low-risk BCC [12,30,31].

IM is believed to have multiple mechanisms of action that are dose-dependent. Initially, within a few hours of application, IM induces destruction through cell necrosis, effectively eliminating tumor keratinocytes at the treatment site. Vascular tumor alteration has also been observed during this stage. Subsequently, within days, this destruction triggers a protein-kinase-dependent immune response that attracts neutrophils, leading to an increase in the antitumoral response through antibody production, along with Toll-like receptor signaling [17,18,31].

IM is marketed for AK as a cream in two concentrations, 0.015% and 0.05%, for use in regimens of two consecutive days on the face/scalp or trunk/extremities, respectively. However, the regimen and concentration for BCC are not yet established. A clinical trial comparing regimens and concentrations has demonstrated a higher clinical and histological response when applying concentrations of 0.05% on consecutive days, with cure rates exceeding 70% and a lower risk of early recurrence, and a better response when the regimen is performed with occlusion. These responses are consistent with subsequent real-world case series showing complete clearances after a single cycle. Favorable acceptance and good patient adherence have also been noted [12,17,31]. Its use has also been tested in nBCC. Several reported cases, although showing less favorable results than with sBCC, position it as an option in cases where surgery is not preferred. In nBCC, a higher risk of scar formation was observed when curettage was performed before applying IM gel. Therefore, it is advisable to wait for the healing of the curettage site before IM application. There are no comparative studies with other therapies in BCC, but in AK, the results compared to 5-FU, imiquimod, and PDT were unfavorable despite good adherence, showing recurrences of over 70% after one year of treatment [31,32].

Regarding adverse effects, IM is well tolerated and has been shown to only produce mild local reactions such as scaling, dryness, and erythema, with severe reactions such as vesicle formation and scarring being rare [12,31].

### 3.5. Electrochemotherapy with Bleomycin

Bleomycin is a cytotoxic agent approved by the FDA and EMA for systemic treatment of various malignancies, including head and neck squamous cell carcinoma (SCC), Hodgkin’s lymphoma, and testicular carcinoma. In dermatology, bleomycin is used off-label to treat vascular malformations, keloid scars, refractory warts, cutaneous leishmaniasis, and various skin tumors such as BCC. Derived from the bacterial species *Streptomyces verticillus*, bleomycin contains glycosylated peptides that disrupt cellular functions and induce DNA damage and tissue growth and repair. Normal tissues express bleomycin hydrolase, conferring resistance to its cytotoxic effects. While skin and lung tissues exhibit lower levels of bleomycin hydrolase, the hydrolase levels in tumor cells remain unknown. Additionally, the hydrophilic nature and large molecular mass of bleomycin pose challenges for transcutaneous delivery. Consequently, intralesional administration in combination with electrochemotherapy (ECT) has emerged as a promising therapeutic strategy [9,33,34].

ECT with bleomycin utilizes electroporation by applying localized short, high-voltage pulses. This process temporarily destabilizes cell membranes, creating pores on the cell surface and enabling enhanced diffusion of bleomycin into tumor cells. This targeted delivery approach aims to reduce and minimize systemic exposure and adverse effects, although the procedure itself can be uncomfortable and painful, requiring anesthesia. Despite its extensive study and application in treating BCCs, its availability is limited in some cases and countries [33,34].

Studies typically followed European Standard Operating Procedures on Electrochemotherapy guidelines. Bleomycin, administered intratumorally or intravenously (or both), was dosed based on tumor and patient characteristics, ranging from 15,000 to 30,000 IU/m^2^ intravenously with adjustments for age and renal function. Intralesional administration used solutions ranging from 250 to 1000 IU/mL. Electric pulse delivery, tailored to BCC characteristics, occurred after 8 or 1 min following intravenous (IV) or intratumoral administration of bleomycin, respectively. Anesthesia varied from local to general based on tumor size, number, and patient characteristics [35].

Meta-analyses and systematic reviews consistently show that ECT demonstrates superior response rates in BCC compared to other cutaneous tumors. Numerous studies, including randomized and uncontrolled clinical trials and case series, have evaluated its exclusive use in BCC, with nBCC or infiltrative BCC being the most studied histological type. While these studies vary in the method of administration, higher response rates have been observed with intratumoral treatment compared to IV administration, although some studies find no differences between the two methods [33,36].

In terms of efficacy, RCTs have reported complete response rates of 86% with ECT, reaching 100% after a second treatment, and maintaining a complete response rate of 89.4% at 5 years of follow-up, with a recurrence rate of 7.5%. Although the recurrence rates are higher than standard surgical treatment, ECT does not show inferiority. Other studies have reported complete response rates ranging from 94% to 100%, with higher rates observed in the intratumoral treatments [33,37].

Regarding side effects, mild adverse reactions such as post-treatment infections, ulceration, erythema, discomfort, nausea, asthenia, localized pain, transient burn marks, and localized necrosis have been commonly observed. Intratumoral administration of ECT generally results in fewer serious adverse effects compared to IV administration. ECT has demonstrated acceptable outcomes in terms of cosmesis and tissue-sparing benefits compared to surgery, although some patients may experience post-treatment hyperpigmentation [33,34,37].

### 3.6. Methotrexate

Methotrexate (MTX) is a widely employed systemic chemotherapy agent, functioning as an antifolate antimetabolite. It acts as a competitive inhibitor of the enzyme folic acid reductase, disrupting the production of thymidylic acid, a crucial pyrimidine metabolite essential for DNA synthesis. Consequently, MTX leads to the arrest of cell division. In the realm of skin cancer treatment, intralesional MTX has showcased notable effectiveness against rapidly proliferating tumors with high mitotic activity, such as keratoacanthoma (KA) and SCC. While concentrations vary between 12.5 and 25 mg/mL, most studies focusing on KA and SCC treatment have adopted weekly doses of 25 mg/mL over 4–6 weeks, resulting in favorable outcomes [7,38].

However, the evidence supporting the effectiveness of intralesional MTX in treating BCC remains scarce. The only published clinical trial to date, which evaluated intralesional MTX in nBCC, did not demonstrate tumor size reduction or histological response, despite using doses similar to those employed for KA. It is plausible that the slower growth rate of BCC tumors may contribute to this limited efficacy compared to KA and SCC. Nevertheless, it is noteworthy that the treatment was well tolerated with no observed local or systemic adverse effects [39].

### 3.7. Interferon Therapy

Interferon (IFN) is a group of cytokines whose mechanism of action is based on stimulating the innate immune response and regulating transcriptional gene activity. These cytokines are divided into three families: Type I IFN, which includes alpha and beta; Type II IFN, which includes the gamma subtype; and Type III IFN, which includes lambda. Both Type I and Type III IFNs appear to elicit a similar response and utilize the same signaling pathways, but their difference lies in their specific response, as they act on receptors in different locations. While there are presently no studies investigating the use of intralesional IFN-lambda in the treatment of NMSC, type I IFNs have been extensively researched for decades [17,40,41,42].

INFs elicit an antitumoral response by activating natural killer cells and macrophages, enhancing lymphocyte cytotoxicity, and increasing the expression of major histocompatibility antigens. Moreover, when BCC is exposed to Type I INF, it triggers the expression of the CD95 receptor, leading to programmed cell death [41].

Notwithstanding the lack of official approval, INF-alpha-2a, INF-alpha-2b, and recombinant INF-beta-1a are commercially available and currently utilized off-label in the management of BCC. Clinical trials have primarily been conducted with alpha-2b, with some involving alpha-2a and recombinant beta-1a. They have been studied in both sBCC and nBCC, employing varied dosing regimens ranging from 1.5 to 30 million units and administered one to three times per week for variable periods of 3–6 weeks. Efficacy rates for INF-alpha range from 52% to 98%, and 67% in the study conducted with beta-1a, all confirmed with proved biopsies [12,17,42,43].

Despite the excellent cosmetic outcomes observed in most studies, the treatment typically requires multiple sessions to achieve a satisfactory response, is costly, and can lead to systemic effects, possibly making it less tolerable than other topical or intralesional therapies. Side effects are dose-dependent and include local reactions and moderate to severe flu-like symptoms such as fever, headache, myalgia, and nausea [12,17,43].

### 3.8. Other Therapies under Investigation (Diclofenac, Calcitriol, Solasodine Glycosides, Sinecatechins, Retinol, Ascorbic Acid, Topical Patidegib, Tirbanibulin, Oncolytic Viral Immunotherapy)

#### 3.8.1. Diclofenac, Calcipotriol, and Combinations

Diclofenac is an FDA-approved non-steroidal anti-inflammatory (NSAID) sodium agent commonly used for AK as a 3% gel twice daily for eight weeks. Apart from its anti-inflammatory properties, it also exhibits antineoplastic characteristics by inducing apoptosis. It functions by inhibiting cyclo-oxygenase-2 (COX-2), which is often overexpressed in epithelial tumors such as AK and other solid tumors, including BCC. This inhibition leads to a reduction in angiogenesis and cellular proliferation [12].

Evidence is limited in the case of BCC, with only one phase II clinical trial demonstrating its potential as a topical treatment for sBCC and no significant changes after treating nBCC. In case of sBCC treated with diclofenac gel in monotherapy, the trial showed a complete histological regression in over 60% of cases with few adverse effects. The most common adverse effect observed was erythema, with occasional instances of swelling, erosions, and pruritus [12,44].

Calcitriol, the biologically active form of vitamin D3, has demonstrated promising antitumor effects in certain solid neoplasms by inhibiting tumor proliferation and activating the vitamin D receptor in epithelial cells. This receptor activation is also believed to exert regulatory control over the hedgehog pathway, thereby enhancing its antiproliferative potential and suggesting a potential role in treating BCC.

In a phase II clinical trial, the efficacy of calcitriol was evaluated both as a 3 μg/g ointment in monotherapy and in combination with 3% diclofenac gel. However, topical calcitriol alone did not demonstrate significant efficacy. Although histological tumor regression was observed when combined with diclofenac, the difference was either negligible or slightly superior to diclofenac monotherapy, with complete histological regression observed in 43.8% of cases. This finding contradicts the anticipated synergy between diclofenac and calcitriol and might be attributed to the counter-proliferative effect demonstrated by calcitriol at low doses. Therefore, exploring different formulations with varying doses and excipients should be considered to optimize treatment strategies.

In line with diclofenac monotherapy, the most common adverse events observed included erythema, both in monotherapy and in the diclofenac/calcipotriol combination, followed by other frequent reactions such as swelling, erosions, and pruritus. Severe reactions were rare and included paresthesias, vesiculation, and scaling [44].

Another combination that has been tested is 3% diclofenac gel combined with 5% imiquimod and ultra-pulsed ablative CO_2_ laser therapy in nodular, infiltrative, and morpheaform BCC. This combination has demonstrated a reduction in the thickness of nBCC but has not shown improvement in infiltrative and morpheaform types [45].

#### 3.8.2. Solasodine Glycoalkaloids

Solasonine and solamargine are glycoalkaloid (GA) derivatives of solasodine, a natural compound found in plants belonging to the Solanaceae family, such as tomatoes, potatoes, eggplants, bell peppers, and chili peppers. They have been extensively used in traditional medicine for treating various disorders with their antitumor properties.

While the exact mechanism of action remains unclear, it is thought that these alkaloids selectively trigger apoptosis and cell death in tumor cells via diverse molecular pathways. They are believed to interact with and disrupt cell membranes, leading to lysis, and that they can also induce the expression of tissue necrosis factor (TNF) receptors within cancer cells, ultimately resulting in apoptosis.

Although GAs have been investigated for their potential as antitumor treatments, research in this field is limited. The initial study, conducted in 1987, assessed their efficacy in treating AK, BCC, and SCC. Low doses of a 0.0005% mixture of solasodine glycosides were applied topically twice daily for 8 weeks, resulting in mild local irritation but complete regression of over 100% of AK and more than 90% of BCC and SCC lesions [46]. Subsequently, in 2008, a controlled clinical trial focusing on morpheaform BCCs demonstrated nearly 70% clearance with low recurrence rates at one year compared to the control group [47]. However, despite these findings, there is insufficient research supporting the topical use of GAs for BCC management, and regulatory agencies have not approved this treatment option. Further studies or case reports validating its efficacy and safety are warranted.

#### 3.8.3. Sinecatechins

Sinecatechins obtained FDA approval in 2006 for its application as a topical treatment for anogenital warts, but since then, its use has been contemplated in the treatment of NMSC. This 10% ointment contains a significantly high concentration of epigallocatechin-3-gallate (EGCG), an active compound derived from green tea.

EGCG has shown its capacity to induce apoptosis in various cancer cells, such as those found in the prostate, lung, gastrointestinal tract, and skin. It is believed that EGCG exerts cytotoxic effects, inhibiting cell growth, and there is speculation that it may play a role in deactivating the Wingless pathway. This pathway’s dysregulation could potentially contribute to the development of BCC, suggesting that EGCG might be considered as a candidate for BCC treatment [12,48].

As of today, sinecatechins have not been established as a standard treatment for skin cancer. Isolated cases have been reported for its use in genital carcinoma associated with the human papillomavirus, and a series of cases have been reported for its use in AK [49,50].

Regarding BCC, only one clinical trial has been conducted to assess its efficacy and tolerance. In this trial, patients applied the ointment twice daily for six weeks, followed by tumor excision after eight weeks. However, no significant difference in histological tumor clearance was observed in these patients compared to those who received only the vehicle. It has been suggested that this may be due to an inadequate amount of EGCG in the ointment or because of a suboptimal formulation. Since numerous studies have demonstrated the antioxidant, anti-inflammatory, and anticarcinogenic effects of EGCG, it has been proposed that its potential role in cutaneous tumors could be more preventive than curative [48].

#### 3.8.4. Retinoid

Retinoids, derivatives of vitamin A, play a crucial role in maintaining skin health by regulating processes such as epidermal turnover, immune responses, and vascularization. Clinical trials have investigated both topical and systemic retinoids as preventive and therapeutic interventions for NMSC.

Although some retinoid treatments have FDA approval, they are not yet standard for keratinocyte carcinomas. FDA-approved topical retinoid treatments include 0.1% topical alitretinoin for cutaneous Kaposi’s sarcoma and oral bexarotene for advanced-stage cutaneous T cell lymphomas. Off-label use of topical retinoids is common for AK, cutaneous T cell lymphomas (CTCL), and in situ SCC [51].

Tretinoin and adapalene are often used off-label for AKs, sometimes combined with other therapies like 5-FU or PDT. Tazarotene shows promise for in situ SCC treatment [51,52].

However, regarding BCC, large-scale trials suggest retinoids are generally ineffective once tumors form. Early studies showed promise with systemic and topical retinoids, but subsequent trials had mixed results [53,54].

Oral retinoids have been studied extensively for BCC chemoprevention, showing potential in reducing new BCC lesions in high-risk populations. However, their significant side effects at effective doses limit widespread use [55].

Etretinate showed better outcomes for AKs and BCCs, with manageable side effects. Topical tretinoin and tazarotene creams have also been explored, showing varying degrees of efficacy.

In conclusion, retinoids are potent dermatological therapies, yet challenges remain in their use for skin cancer prevention and treatment. These include limited testing of novel compounds like trifarotene, gaps between research findings and clinical applications, and concerns about side effects. Addressing these challenges through further research and precision medicine approaches can enhance the future use of retinoids in prevention and treatment of NMSC [51].

#### 3.8.5. Ascorbic Acid

Ascorbic acid (AA) has a lengthy and debated history as an anti-cancer agent. A recent study conducted by Burke and colleagues compared the effectiveness of 30% ascorbic acid with 5% topical imiquimod in treating BCC. Topical ascorbic acid demonstrated superiority at 8 weeks and was equally effective at 12 weeks compared to topical imiquimod for low-risk lesions. Additionally, ascorbic acid was associated with fewer adverse effects than imiquimod [56].

#### 3.8.6. Topical Patidegib

Recently, novel therapies have emerged for advanced and metastatic cases but also to seek therapies that avoid surgery in patients with Gorlin syndrome, as well as in those with multiple sporadic BCCs, acting upon the hedgehog pathway by inhibiting the Transmembrane Smoothened Protein (SMO). In addition to systemic options like vismodegib and sonidegib, researchers have also explored the development of topical inhibitors. Among these, topical patidegib has shown promise in diminishing tumor burden, potentially mitigating the side effects associated with systemic targeted treatments [57]. In the trials, the regimen has been using both 2% and 4% concentrations, both tested once daily and twice daily for two weeks. Currently, efficacy and safety studies with a dose of 2% twice daily for one year are ongoing, awaiting results (NCT03703310, NCT04308395).

Despite positive preliminary results for BCC treatment [58], the latest data do not demonstrate histological clearance of the skin lesions. The lack of response appears to be associated with inadequate drug penetration coupled with an insufficient biological response [59].

#### 3.8.7. Tirbanibulin

Tirbanibulin 1% ointment is a synthetic antiproliferative agent approved by the FDA in 2020 for treating AK located on the face or scalp [60]. Two cases have been documented in the literature regarding the utilization of tirbanibulin in BCC, yielding promising outcomes [61,62]. Further research involving larger cohorts is required to validate the efficacy of this therapy in treating BCC.

#### 3.8.8. Oncolytic Viral Immunotherapy

Oncolytic viruses have emerged as promising agents in cancer treatment. They target specific tissues and are currently undergoing investigation in clinical trials. The potential antitumoral therapies being explored encompass DNA and RNA viruses, including herpes simplex virus type 1 (HSV-1) and adenovirus, among others. These oncolytic viruses are modified by inserting immunomodulatory transgenes into the viral genome, thereby effectively enhancing antitumoral immunity. They can express reporter genes, cytokines (such as granulocyte-macrophage colony-stimulating factor (GM-CSF), interleukin-12, or interferon (IFN)), as well as chemokines, immune checkpoint inhibitors, co-stimulatory checkpoints, bispecific T-cell engagers, tumor-associated antigens, and combinations thereof. Additionally, they may be combined with other immunomodulatory agents such as chemotherapy or radiotherapy to enhance their efficacy [63,64].

1. Intralesional Talimogene Laherparepvec (T-VEC)

T-VEC stands as a groundbreaking oncolytic viral therapy, being the first treatment approved by both the FDA and EMA for locally unresectable recurrent melanoma after initial surgery. Derived from genetically modified HSV-1 expressing GM-CSF, T-VEC showcases dual capabilities, augmenting both local and systemic antitumor immune responses. Upon intratumoral administration, T-VEC initiates direct cytotoxic effects on tumor cells, resulting in their destruction. The incorporation of the human GM-CSF gene enhances the recruitment and activation of dendritic and macrophages, thus intensifying the immune-mediated assault on cancer cells [65,66].

Despite the lack of scientific evidence regarding its efficacy and safety in NMSC, ongoing clinical trials are currently investigating its potential use in this context. These trials include a Phase I study evaluating locally advanced SCC, BCC, Merkel cell carcinomas, and cutaneous T-cell lymphomas (NCT03458117), as well as a Phase II trial involving T-VEC with nivolumab for refractory NMSC, which encompasses BCC (NCT02978625) [57]. Recruitment is also underway for the study in transplant patients and advanced cutaneous cancer (NCT04349436) [2].

2. IFN gamma Adenovirus

Another oncolytic viral therapy currently under investigation for treating BCC is the adenovirus encoding human IFN-gamma, known as ASN-002. This replication-defective adenovirus vector expresses the recombinant human IFN-gamma gene, demonstrating potential in inhibiting cell proliferation and inducing immune-mediated antitumor effects. A clinical trial has been completed to assess the safety, tolerability, and preliminary efficacy of ASN-002 in relapsed or refractory lymphoma and advanced solid tumors. Moreover, research is ongoing to explore its application in dermatology, aiming to enhance immune-mediated responses in non-oncological conditions such as atopic dermatitis and chronic eczema [57,65].

Two trials are underway to assess the efficacy and safety of IFN gamma Adenovirus in treating BCC. One trial, which investigated ASN-002 combined with 5-FU, has concluded, but its results are pending publication (NCT02550678). Another trial is currently recruiting participants to evaluate ASN-002 in combination with vismodegib (NCT04416516) [57,65].

#### 3.8.9. Novel Therapies

Several novel topical and intralesional therapies are currently under investigation, showing promise in advancing the treatment options for BCC and potentially improving patient outcomes:

1. Topical Remetinostat: Remetinostat inhibits histone deacetylase (HDAC), altering gene expression to suppress tumor growth. A Phase 2 trial (NCT03180528) recently completed examined the efficacy of topical remetinostat gel 1%, applied three times daily for 6 weeks.
2. Intralesional IFx-Hu2.0 and STP705: IFx-Hu2.0 is a pDNA-encoding Emm55 autologous cancer cell vaccine that stimulates the immune system to recognize and target BCC cells expressing the Emm55 antigen, promoting tumor cell destruction. STP705 is a siRNA nanoparticle targeting TGF-β1 and COX-2, which silences expression of TGF-β1 and COX-2, inhibiting tumor growth and reducing inflammation within the BCC microenvironment. Both have been examined as intralesional treatments for BCC in phase II trials, which have already been completed (NCT04925713 and NCT04669808).
3. Intralesional Cemiplimab: Cemiplimab blocks the PD-1 receptor, enhancing the immune response against BCC cells, leading to tumor regression. There is one phase 1 trial currently recruiting patients to evaluate the anti-PD-1 antibody response in both BCC and cutaneous SCC (NCT03889912).
4. Intralesional L19IL2/L19TNF (Daromun/Fibromun): L19IL2/L19TNF targets fibronectin’s extra-domain B in the tumor microenvironment, delivering IL-2 or TNF-α to stimulate the immune response and induce tumor cell death. Two phase 2 trials are recruiting patients to investigate their use in BCC (NCT04362722 and NCT05329792) [2,57].

## 4. Conclusions

In summary, the management of BCC has witnessed significant advancements, particularly in the realm of non-surgical therapies. Topical and intralesional immunotherapies have emerged as promising alternatives, particularly for low-risk sBCC and select cases of nBCC [2]. A summary of these is presented in Table 1.

Imiquimod, 5-FU, and PDT have demonstrated efficacy in achieving histological clearance, with imiquimod showing superiority over PDT and similarity to 5-FU in treating sBCC [10,16] PDT presents excellent cosmetic outcomes and has shown promise, particularly in lesions on the lower limbs and in older patients [12]. Intralesional PDT has emerged as a novel approach, demonstrating non-inferiority to surgery, and just with a single session [28]. IM gel has shown efficacy in experimental studies and isolated cases of superficial and low-risk BCC, but further research is needed to establish optimal regimens and concentrations [31]. ECT with bleomycin has demonstrated superior response rates compared to other cutaneous tumors, with acceptable outcomes in terms of cosmesis and tissue-sparing benefits [33]. While methotrexate and interferon therapy have shown limited efficacy in BCC treatment, further investigation is warranted to elucidate their role in this context [38,41].

Moreover, ongoing research into novel therapies such as diclofenac, calcitriol, solasodine glycosides, sinecatechins, retinoids, ascorbic acid, topical patidegib, tirbanibulin, and oncolytic viral immunotherapy offers promising avenues for the future of BCC management [2,46,49,51,56,58,63,65]. These therapies, although at varying stages of development and clinical validation, provide additional options for patients who are not candidates for or prefer alternatives to surgery. Overall, the expanding armamentarium of non-surgical therapies offers valuable alternatives for patients with low-risk BCC or those contraindicated for surgery, emphasizing the importance of individualized treatment approaches tailored to patient and tumor characteristics. Further research and clinical trials are essential to refine treatment protocols, optimize outcomes, and expand the therapeutic options available for BCC management. It is important to recognize that this therapy might be of potential usefulness in both neoadjuvant and adjuvant settings in specific situations.

## 5. Limitations

Among the weaknesses of this review, it is worth noting the lack of a systematic search strategy, which consequently increases the risk of omitting relevant studies and introducing biases into the review. Also, this review is limited by the fact that we could only review properly Spanish and English language articles. Additionally, the heterogeneity among the studies should be highlighted. This can complicate data synthesis and interpretation, potentially weakening its robustness. Furthermore, such heterogeneity limits the generalizability of the findings of this review in their applicability to the management of BCC in routine clinical practice.

## Figures and Tables

**Table 1:** ALA: 5-aminolevulinic acid; MAL: methyl-aminolevulinate; MHC: Major Histocompatibility Complex; PDT: Photodynimic therapy;INF: Interferon; COX-2: Cyclooxigenase-2 TNF: Tumor necrosis factor; EGCG: epigallocatechin-3-gallate; T-VEC: Talimogene Laherparepvec; HSV-1: Herpes simplex virus type I; GM-CSF: Granulocyte-Macrophage Colony-Stimulating Factor.

Treatment	Usage Classification	Mechanism of Action	Dosage and Route of Drug Administration	Clearance Rate (SBCC)	Adverse Effects
IMIQUIMOD [7,8,9,10,11,12]	Approved	Stimulating Toll-like receptors 7 and 8, triggering a type 1 helper T cell cytokine response	5% cream Five times a week for six weeks Topical	75.2%	Common: Local skin reactions (erythema, scabbing, itching, pain, and tenderness) (28.1%)
5-FLUOROURACIL [6,9,12,13,14,15,16,17,18,19,20,21,22,23]	Approved	Disrupt DNA and RNA synthesis by inhibiting thymidylate synthetase enzyme and induce cell death	5% cream Twice daily for 2–12 weeks Topical	90%	Common: Local skin reactions (erythema, scabbing, itching, pain, and tenderness) (62–99%)
	Of-label		0.5 to 2 mL at concentrations between 15 and 50 mg/mL 1–3 times weekly for 2–6 weeks. Intradermal	90% at 9–12 weeks	Common: local and transient skin reactions Rare: Systemic effects (cytopenias and gastrointestinal disturbances)
PHOTODYNAMIC THERAPY (ALA AND MAL) [2,6,12,16,17,24,25,26,27]	Approved	Photosensitizers gather in fast-growing cells, converting into protoporphyrin IX. When exposed to red light, protoporphyrin IX creates cytotoxic oxygen species, causing cell destruction	Two sessions of PDT with 20% MAL cream, spaced one week apart Consider new cycles if there is clinical/histological persistence Topical	60–100%	Very common: itching and pain during radiation Common: Local inflammatory reactions (erythema, erosions, crusts) Rare: Hyperpigmentation, tissue necrosis, or scarring
INGENOL MEBUTATE [12,17,18,28,29,30]	Off-label	Eradicates keratinocytes by inducing cell death at the application site + triggers an immune response by stimulating antibody production	0.05% cream Once daily for two consecutive days Topical	70% Better outcomes with occlusion	Common: Local skin reactions (erythema, scaling, dryness) Rare: vesicle formation and scarring
ELECTROCHEMOTHERAPY WITH BLEOMYCIN [9,31,32,33,34,35]	Off-label	Disrupt cellular functions and induce DNA damage and tissue growth and repair	Solutions range from 250 to 1000 IU/mL. Electric pulse delivery after 1 min of intralesional administration Intradermal	86–100% higher rates observed in the intralesional treatments and after a 2nd cycle	Common: post-treatment infections, ulceration, erythema, discomfort, nausea, asthenia, pain, burn marks, necrosis Rare: hyperpigmentation
METHOTREXATE [7,36,37]	Off-label/Under investigation	Disrupts DNA synthesis by inhibiting folic acid reductase, leading to the cessation of cell division	Weekly doses of 12.5–25 mg/mL over 4–6 weeks Intradermal	Limited evidence, with unfavorable outcomes	Limited evidence, no local or systemic adverse effects were observed
INTERFERON (ALPHA-2A, ALPHA-2B, AND RECOMBINANT BETA-1A) [12,17,38,39,40,41]	Off-label	Activates natural killer cells and macrophages, enhances lymphocyte cytotoxicity, increases MHC antigen expression and triggers CD95 receptor expression, leading to programmed cell death	1.5 to 30 M UI 1–3 times per week for variable periods of 3–6 weeks Intradermal	INF-alpha: 52–98%, INF-beta-1a: 67%	Common: local skin reactions and moderate to severe flu-like symptoms (fever, headache, myalgia, nausea)
DICLOFENAC [12,42,43]	Off-label	Inhibiting overexpressed COX-2 in epithelial tumors, leading to a reduction in angiogenesis and cellular proliferation	3% gel twice daily for eight weeks Topical	60%	Local skin reactions (erythema, swelling, scaling, itching)
SOLASODINE GLYCOALKALOIDS [44,45]	Off-label (Research in this field is limited)	Interact with and disrupt cell membranes, leading to lysis, and that they can also induce the expression of TNF receptors within cancer cells, ultimately resulting in apoptosis.	0.0005% mixture of solasodine glycosides cream, applied twice daily for 8 weeks Topical	70–90% (Limited evidence)	Common: mild local irritation (Limited evidence)
SINECATECHINS (EGCG) [12,46,47,48]	Off-label (Research in this field is limited)	Cytotoxic effects, inhibiting cell growth. it may play a role in deactivating the Wingless pathway	10% ointment twice daily for six weeks No standard treatment stablished Topical	Limited evidence, with unfavorable outcomes. Possible preventive role	-
RETINOID [49,50,51,52,53]	Off-label/Under investigation	Epidermal turnover, immune response, and vascularization regulation	No set doses or guidelines yet	Mixed results. Possible preventive role	-
ASCORBIC ACID [54]	Off-label/Under investigation	Antioxidant that prevents tissue damage caused by free radicals. Debated efficacy as an antineoplastic agent.	30% cream Twice daily for 8 weeks Topical	13/15 (86.7%)	Common: Local skin reactions (erythema, scaling, erosions)
TOPICAL PATIDEGIB [55,56,57]	Under investigation	Hedgehog pathway inhibition	2% gel Twice daily for two weeks Topical	0%	-
TIRBANIBULIN [58,59,60]	Under investigation	Disrupts microtubule network and inhibits cell division	Once daily for five consecutive days Topical	-	Common: Local skin reactions, (erythema, pain or burning sensation)
T-VEC [2,55,61,62,63,64]	Under investigation	Genetically modified HSV-1 expressing GM-CSF, with direct cytotoxic effects on tumor cells, resulting in cell destruction	- Intradermal	-	-
IFN GAMMA ADENOVIRUS [55,61,62,63]	Under investigation	Genetically modified adenovirus encoding human IFN-gamma, inhibits cell proliferation and induces immune-mediated antitumor effects	- Intradermal	-	-
